# Inducible and constitutive heat shock gene expression responds to modification of *Hsp70 *copy number in *Drosophila melanogaster *but does not compensate for loss of thermotolerance in *Hsp70 *null flies

**DOI:** 10.1186/1741-7007-6-5

**Published:** 2008-01-22

**Authors:** Brian R Bettencourt, Catherine C Hogan, Mario Nimali, Brian W Drohan

**Affiliations:** 1 Department of Biological Sciences, University of Massachusetts Lowell, 1 University Avenue, Lowell, MA 01854, USA; 2 Department of Computer Science, University of Massachusetts Lowell, 1 University Avenue, Lowell, MA 01854, USA

## Abstract

**Background:**

The heat shock protein Hsp70 promotes inducible thermotolerance in nearly every organism examined to date. Hsp70 interacts with a network of other stress-response proteins, and dissecting the relative roles of these interactions in causing thermotolerance remains difficult. Here we examine the effect of *Hsp70 *gene copy number modification on thermotolerance and the expression of multiple stress-response genes in *Drosophila melanogaster*, to determine which genes may represent mechanisms of stress tolerance independent of Hsp70.

**Results:**

*Hsp70 *copy number in four strains is positively associated with *Hsp70 *expression and inducible thermotolerance of severe heat shock. When assayed at carefully chosen temperatures, *Hsp70 *null flies are almost entirely deficient in thermotolerance. In contrast to expectations, increasing *Hsp70 *expression levels induced by thermal pretreatment are associated with increasing levels of seven other inducible *Hsps *across strains. In addition, complete *Hsp70 *loss causes upregulation of the inducible *Hsps *and six constitutive stress-response genes following severe heat shocks.

**Conclusion:**

Modification of *Hsp70 *copy number quantitatively and qualitatively affects the expression of multiple other stress-response genes. A positive association between absolute expression levels of *Hsp70 *and other *Hsps *after thermal pretreatment suggests novel regulatory mechanisms. Severe heat shocks induce both novel gene expression patterns and almost total mortality in the *Hsp70 *null strain: alteration of gene expression in this strain does not compensate for *Hsp70 *loss but suggests candidates for overexpression studies.

## Background

The heat shock protein Hsp70 is a fundamental molecular mechanism of inducible thermotolerance, but it does not act alone. Genetic, biochemical and physiological analyses in *Drosophila *and numerous systems establish Hsp70's central role [1-3]. However central, Hsp70 must work as part of an interactive network of other Hsps: some inducible, some constitutive [4-7]. Transgenic studies have established that specific modification of Hsp70 expression alters thermotolerance in cells and organisms [1,8-10]. Still, the degree to which Hsp70 itself generates stress-tolerance phenotypes, versus the interactions of Hsp70 with other Hsps, remains difficult to tease apart.

Such dissection is necessary for research in the prophylactic stress protection of cells, tissues and organisms to progress. Ideal candidate genes for manipulation will maximize protection with minimal cost to the organism. *Hsp70 *is a poor candidate: although induction of Hsp70 is protective of future stress, in the absence of such a stress, Hsp70 is deleterious [11,12]. Thus, the challenge is to identify genes that promote stress tolerance in the *absence *of Hsp70, a task made difficult by Hsp70's critical position in the stress-response network. Finding these genes demands simple genetic tools to disrupt Hsp70's role in the stress response, and better understanding of the richness of the network itself. Beyond identification of genes whose expression is modified by stress, analysis of stress-by-Hsp70-by-gene expression interactions is required.

Gong and Golic [13,14] provided a key toolkit by specifically deleting the *Hsp70 *genes from the *Drosophila melanogaster *genome, producing complete and partial knockout strains. In this study we employ two of these strains: one lacks all *Hsp70*s, the other lacks half (*Hsp70Aa*, *Hsp70Ab *and *Hsp70Ba*; see the methods section). The deletion strains are an ideal counterpart to the extra-*Hsp70 *flies produced by Welte et al [15] and allow direct measurement of *Hsp70*'s contribution to thermotolerance and the stress-response network. In addition, whole-genome expression analyses have dissected the inducible heat shock transcriptional response in wild-type and mutant flies. Sorensen et al [16] established that beyond rapid Hsp induction, thermal stress upregulates and downregulates multiple *Hsp *and non-*Hsp *genes in at least three temporally distinct expression clusters. Neal et al [17] applied a similar approach to examining flies whose heat-inducible transcriptional response was ablated by mutation in HSF (heat shock transcription factor). Disruption of HSF activity at heat shock elements in *Hsp *gene promoters eliminates much stress-inducible *Hsp *expression (including, but not exclusively, that of *Hsp70*), but increases inducible expression of *Hsp40 *and *Hsp83*.

Both *Hsp70 *and *HSF *mutant flies maintain some degree of thermotolerance [14,17]. Gong and Golic suggest that compensatory modification of both constitutive and inducible heat shock gene expression (i.e. *Hsc70-4 *and *Hsp68*) may underlie the maintenance of thermotolerance. Neal et al [17] report that in addition to *Hsp40 *and *Hsp83*, the glutathione S-transferase *GstE1 *is induced by heat shock in *HSF *mutant flies. These studies suggest that elimination of Hsp70/HSF uncovers additional compensating mechanisms of thermotolerance and exacerbates them. Perturbing the participation of Hsp70 in the Hsp network may thus expose and modify the expression profiles of genes such as *Hsc70-4*, *Hsp40*, *Hsp68*, *Hsp83 *and *GstE1 *that promote thermotolerance when Hsp70 cannot. Recent work also assigns key stress-protective roles to *Drosophila *inducible small Hsps such as Hsp22 and Hsp27 [18-20] and constitutive Hsc70s [21-23], whose functional independence from Hsp70 remains poorly understood. These and additional candidate stress-protective genes could protect organisms from future stress, with lower costs than Hsp70.

In this study, we examined the expression of genes sensitive to stress and Hsp70 modification in a panel of *Hsp70 *mutant strains undergoing thermal stress. We designed critical stresses to maximize the 'signals' of Hsp70 manipulation (differences in Hsp70 expression and thermotolerance) because uncovering mechanisms of compensation for Hsp70 loss requires establishing the precise modes of thermotolerance that Hsp70 controls. Thus, we first sought to identify lesions in thermotolerance that differentiated *Hsp70 *null, underexpressing, wild-type and overexpressing flies, to determine whether *Hsp70 *null flies would be quantitatively or qualitatively different. Next, we measured the effects of *Hsp70 *manipulation on expression of constitutive and inducible stress-response genes during the critical stresses. We sought to determine whether multiple genes would respond coordinately, whether constitutive versus inducible genes would respond similarly and, finally, how genetic modification of Hsp70 expression modifies stress-by-gene expression interactions. Thus, we ask the question: if compensation for Hsp70 loss truly exists, do its genetic bases include alteration of stress gene expression?

## Results

### Treatments

Based on preliminary experiments, we designed and applied the following treatments in analyses of thermotolerance and gene expression: 'C', 3 h at 22°C; 'PT', 1 h at 36°C followed by 2 h at 22°C; 'HS39', 'HS39.5', 2 h at 22°C followed by 1 h at 39 or 39.5°C; 'PT+HS39', 'PT+HS39.5', 1 h at 36°C, followed by 1 h at 22°C, followed by 1 h at 39 or 39.5°C. See the methods section for a detailed description.

### Thermotolerance

**Figure 1:**
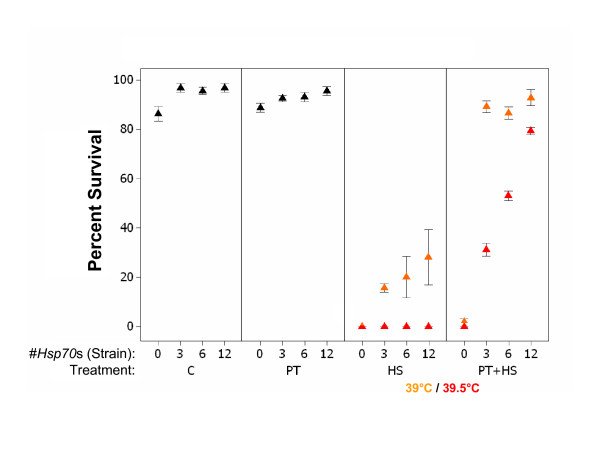
**Variation in larval thermotolerance**. The *y*-axis displays the percentage of third-instar larvae that survived to adulthood after control, pretreatment, heat shock and pretreatment plus heat shock treatments (C, PT, HS and PT+HS respectively on the *x*-axis). '#*Hsp70*s (Strain)' indicates *Hsp70 *copy numbers of the *Hsp70*^-^, *Hsp70*^*A*-*Ba*-^, *Hsp70*^+^and *Hsp70*^*traIII *^strains, respectively. Symbols are means ± 1 standard error (SE). For HS and PT+HS treatments, orange symbols indicate heat shocks of 39°C and red symbols indicate heat shocks of 39.5°C. See the methods section for a full description of the treatments.

Variation in larval thermotolerance among the strains is highly significant according to binary logistic regression, and extremely sensitive to temperature (Table 1 and Figure 1). *Hsp70*^- ^larvae experience an approximate 10% reduction in survival following C treatment, while the other strains do not. Thermal pretreatment marginally decreases survival, with the effect inversely related to *Hsp70 *copy number. The haploid genomic *Hsp70 *copy number of the strains we examined is: *Hsp70*^-^, 0; *Hsp70*^*A*-*Ba*-^,3;*Hsp70*^+^, 6;*Hsp70*^*traIII*^, 12 [13,15]. All strains have zero basal thermotolerance of 39.5°C (HS). In contrast, basal thermotolerance of 39°C is positively associated with *Hsp70 *copy number (strain), and ranges from 1 to 30% (Figure 1). A separate logistic regression of survival of HS39 on *Hsp70 *copy number is significant (*G *= 72.511, *P *< 0.001).

**Table 1:** Binary logistic regression of thermal stress survival (larval survival to adulthood) on strain, treatment and strain-by-treatment interaction

Term	χ^2^	df
Strain	169.356	3
Treatment	222.070	5
Strain-by-treatment	54.871	15

Strains: *Hsp70*^-^, *Hsp70*^*A*-*Ba*-^, *Hsp70*^+^, *Hsp70*^*traIII*^. Treatments: C, PT, HS39, HS39.5, PT+HS39 and PT+HS39.5 as described in the text. Log-Likelihood = -1305.466. All χ^2 ^values significant at *P *< 0.001. Test that all slopes are zero: *G *= 3095.978, degrees of freedom (df) = 23, *P*-value *P *< 0.001.

Inducible thermotolerance (PT+HS) is more strongly affected by *Hsp70 *copy number. At 39°C, the *Hsp70*^- ^displays 2% induced survival, while all other strains are indistinguishable at 86–95%. At 39.5°C, inducible thermotolerance ranges from 0 to 75% and is positively associated with *Hsp70 *copy number in a strongly linear fashion (Figure 1). Logistic regression of survival of PT+HS39.5 on *Hsp70 *copy number is significant (*G *= 283.295, *P *< 0.001).

### Analysis of gene expression

We analyzed quantitative real-time PCR (qrtPCR) data according to Montooth et al [24]: Expression is measured as a reciprocal of critical threshold (1000/CT, where 'CT' is the number of cycles required for reactions to yield fluorescence above background). Briefly, following univariate tests of normality, mixed analysis of variance (ANOVA) models were fitted to expression values using maximum likelihood estimation and the SAS software suite (SAS Institute). Mixed models included random effects of strain, treatment, timepoint, extraction replicate and polymerase chain reaction (PCR) plate and replicate. Expression of the ribosomal-protein gene *RpL32 *was included as a continuous covariate of candidate gene expression level, to account for variation in RNA/cDNA preparation. Thus, least square mean (LSM) estimates reported by the ANOVA reflect each gene's expression level when controlling for variation in *RpL32*, and are reported in natural units accordingly (mean reciprocal CTs, rather than experimental gene:*RpL32 *ratios). *RpL32 *expression was significantly affected by treatment (ANOVA; *P *< 0.0001); specifically, HS and PT+HS treatments reduced *RpL32 *expression in all strains, likely reflecting overall reduction in non-heat shock gene transcription during severe thermal stress [25,26]. However, these treatments did not reduce the correlation between *RpL32 *and other genes: *RpL32 *is a significant predictor of candidate gene expression in all gene/timepoint combinations but three (Table 2). See the discussion section for further consideration of raw versus modeled gene expression data and the implications of variation in control gene expression under experimental treatments.

**Table 2:** *F*-statistics and statistical significance of mixed model ANOVAs of gene expression

Gene	Time	Strain	Treatment	Strain-by-treatment	*RpL32*
*GstE1*	0	13.27****	3.25*	3.15**	12.36***
	1	6.05**	11.42****	6.04****	8.13**
*Hsc70_1*	0	†	†	†	†
	1	†	†	†	†
*Hsc70_2*	0	9.79****	9.96****	4.39****	27.36****
	1	7.74***	18.63****	7.11****	6.58*
*Hsc70_3*	0	2.4	11.44****	12.8****	20.82****
	1	6.28**	53.46****	11.45****	14.68***
*Hsc70_4*	0	7.71***	33.5****	17.83****	25.62****
	1	6.66***	51.26****	20.17****	21.47****
*Hsc70_5*	0	4.42**	17.69****	7.08****	27.46****
	1	10.84****	34.83****	13.34****	17.76****
*Hsp22*	0	8.32***	50.25****	7.89****	7.77**
	1	4.41**	75.47****	11.81****	9**
*Hsp23*	0	4.33**	17.26****	7.92****	7.37**
	1	10.41****	50.16****	18.68****	7.77**
*Hsp26*	0	2.78	39.13****	4.38****	14.83***
	1	1.32	45.33****	7.97****	1.9
*Hsp27*	0	2.32	14.5****	10.7****	24.39****
	1	3.77*	52.41****	17.26****	20.94****
*Hsp40*	0	2.12	25.39****	13.65****	19.15****
	1	3.22*	70.16****	23.96****	11.17**
*Hsp60*	0	3.26*	28.6****	14.89****	0.64
	1	20.72****	81.57****	19.84****	19.25****
*Hsp67Ba*	0	1.23	2.05	1.76	25.24****
	1	0.83	4.62**	2.11*	17.54****
*Hsp67Bb*	0	‡	‡	‡	‡
	1	‡	‡	‡	‡
*Hsp67Bc*	0	2.16	11.82****	7.37****	15.99***
	1	4.44	38.33****	12.58****	25.98****
*Hsp68*	0	1.3	45.67****	12.28****	45.1****
	1	3.02*	82.17****	18.6****	30.48****
*Hsp70*	0	49.74****	43.36****	11.57****	11.84**
	1	70.62****	89.25****	13.64****	4.2*
*Hsp83*	0	1.19	16.64****	12.54****	28.63****
	1	4.44**	53.35****	14.05****	3.41

'Gene' and 'Time' indicate gene examined and time following treatment, respectively. Remaining columns indicate *F*-statistics associated with effects of Strain (df = 3), Treatment (df = 5), strain-by-treatment interaction (df = 15), and expression of the *RpL32 *control gene (covariate; df = 1). Significance levels: ****, *P *< 0.0001; ***, *P *< 0.001; **, *P *< 0.01; *, *P *< 0.05. Cells without asterisks indicate statistically insignificant values. † indicates that *Hsc70-1 *was not amplified with sufficient efficiency to calculate a standard curve and was excluded from analysis. ‡ indicates that *Hsp67Bb *was not amplified with sufficient specificity and was excluded from analysis.

**Figure 2:**
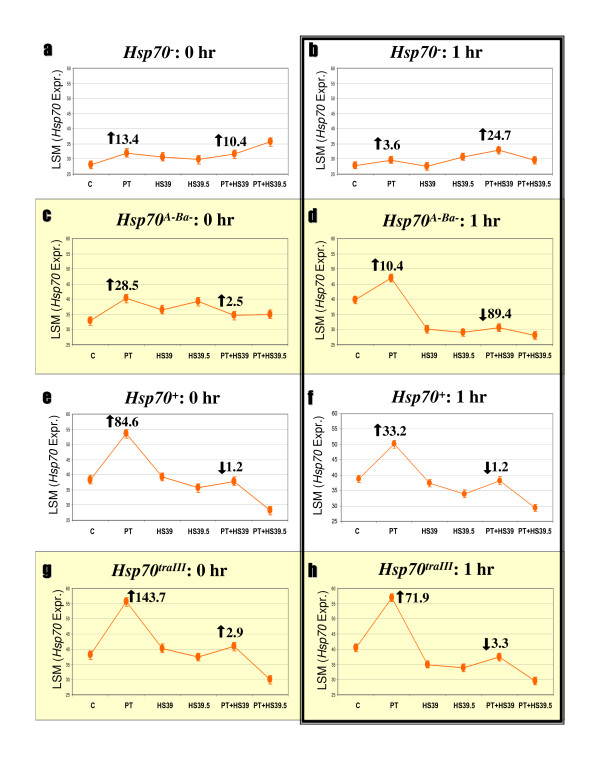
**Variation in *Hsp70 *expression**. Each graph displays LSM estimates of *Hsp70 *gene expression following C, PT, HS39, HS39.5, PT+HS39 and PT+HS39.5 treatments (see the methods section for a full description of the treatments). LSMs are expressed in reciprocally transformed CT values (1000/cycle number). Symbols are means ± 1 SE. Graphs are organized according to strain (left to right) and timepoint post treatment (top to bottom). Fold changes in expression levels after PT and PT+HS39, relative to C, are indicated by the large arrows and numbers; arrows indicate direction of change.

**Figure 3:**
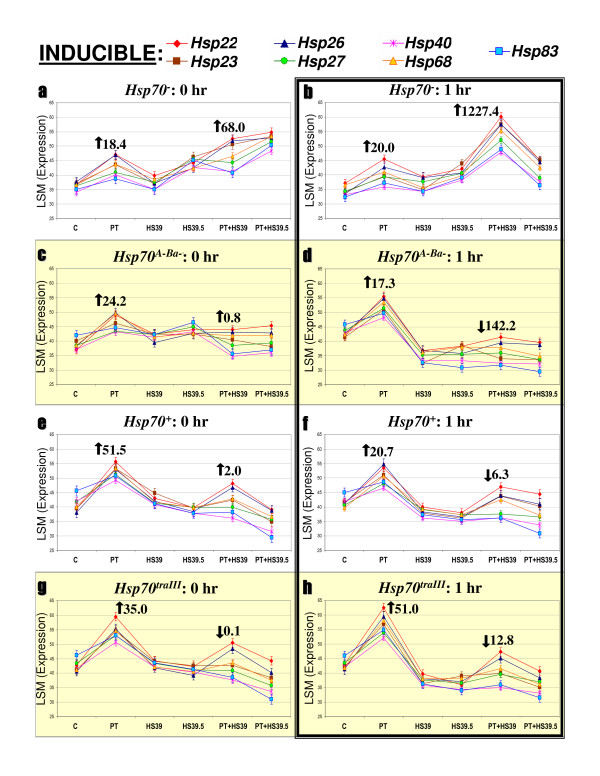
**Variation in inducible *Hsp *gene expression**. Each graph displays LSM estimates of seven genes' expression following C, PT, HS39, HS39.5, PT+HS39 and PT+HS39.5 treatments (see the methods section for a full description of the treatments). LSMs are expressed in reciprocally transformed CT values (1000/cycle number). Symbols are means ± 1 SE; the legend at top of figure indicates gene-symbol pairs. Graphs are organized according to strain (left to right) and timepoint post treatment (top to bottom). Mean fold changes in expression levels of all seven genes after PT and PT+HS39, relative to C, are indicated by the large arrows and numbers; arrows indicate direction of change.

**Figure 4:**
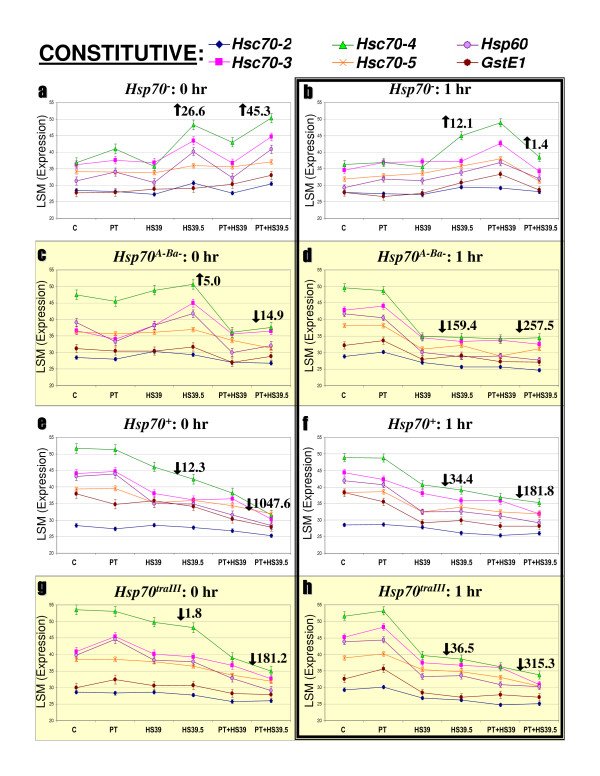
**Variation in constitutive gene expression**. Each graph displays LSM estimates of six genes' expression following C, PT, HS39, HS39.5, PT+HS39 and PT+HS39.5 treatments (see the methods section for a full description of the treatments). LSMs are expressed in reciprocally transformed CT values (1000/cycle number). Symbols are means ± 1 SE; the legend at top of figure indicates gene-symbol pairs. Graphs are organized according to strain (left to right) and timepoint post treatment (top to bottom). Mean fold changes in expression levels of all seven genes after HS39.5 and PT+HS39.5, relative to C, are indicated by the large arrows and numbers; arrows indicate direction of change.

We analyzed expression at zero and one hour post-treatment separately for simplicity; when included as an effect in a combined ANOVA, timepoint, timepoint by strain and timepoint by treatment interactions were highly significant for more than 75% of genes examined (data not shown). We measured fold changes in expression in two ways: within strains and between strains. In both cases, fold changes are calculated as reaction efficiencies raised to the power of differences between LSM (non-reciprocal) CT values [27]. Within strains, we measured fold changes in gene expression after heat treatments relative to control treatment (e.g. PT relative to C, PT+HS relative to C, see Figures 2, 3 and 4). Between strains, we measured fold changes in gene expression within treatments and timepoints (as described in the following).

### *Hsp70 *expression

Low levels of apparent *Hsp70 *products were amplified from *Hsp70*^- ^cDNA. After most treatments, levels of *Hsp70 *amplification in the *Hsp70*^- ^strain are orders of magnitude lower (Table 3, Figure 2). For example, *Hsp70 *levels in the *Hsp70*^- ^strain immediately after C treatment are 17.4-, 472.6- and 411.7-fold lower than the *Hsp70*^*A*-*Ba*-^, *Hsp70*^+ ^and *Hsp70*^*traIII *^strains after C treatment, respectively. Immediately after PT treatment, levels are 140.5-, 414262- and 1515423-fold reduced (Table 3).

**Table 3:** Fold changes in *Hsp70 *expression in the *Hsp70*^*A*-*Ba*-^, *Hsp70*^+ ^and *Hsp70*^*traIII *^strains relative to the *Hsp70*^- ^strain, at zero and one hour post treatment

		**Treatments**					
		
Time	Strain	C	PT	HS39	HS39.5	PT+HS39	PT+HS39.5
0 h	*Hsp70*^*A*-*Ba*-^	17.36	140.46	32.97	-8.46	6.18	-1.41
	*Hsp70*^+^	472.59	414262.26	172.32	33.28	43.58	-88.42
	*Hsp70*^*traIII*^	411.68	1515422.52	311.33	97.15	278.87	-30.26
1 h	*Hsp70*^*A*-*Ba*-^	1164.28	1.24	4.41	-2.82	-3.81	-2.34
	*Hsp70*^+^	669.44	205466.66	363.09	7.04	26.10	-1.09
	*Hsp70*^*traIII*^	1939.22	12930136.23	83.16	6.59	16.36	1.08

Treatments are as described in the text.

**Figure 5:**
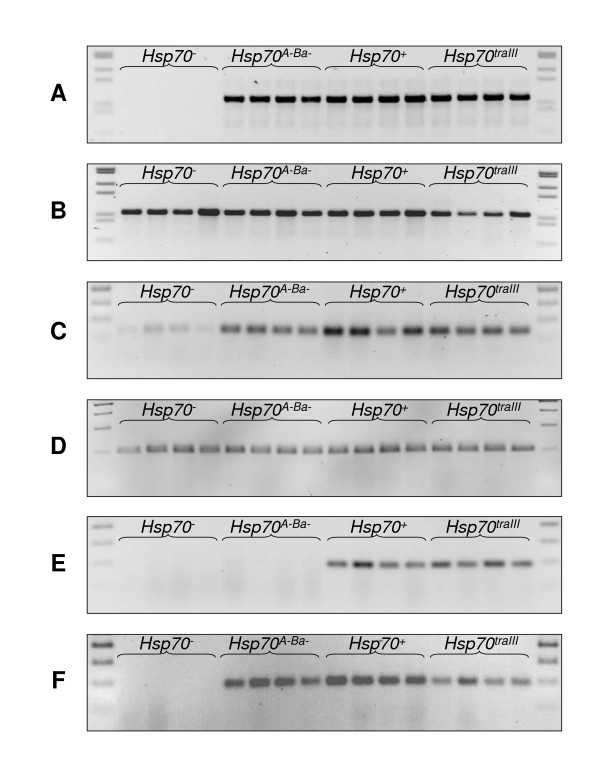
**Gel electrophoresis of PCR products amplified from individual fly genomic DNAs**. In each gel, DNA size standards are in outermost lanes, interior lanes contain products obtained from four individual *Hsp70*^-^, *Hsp70*^*A*-*Ba*-^, *Hsp70*^+ ^and *Hsp70*^*traIII *^flies as marked. (A) 1 kb *Hsp70 *fragment, comprising the 3' half of the *Hsp70 *CDS. (B) 1 kb *fd3F *fragment. (C) 140 bp *Hsp70 *fragment, from the 3' end of the CDS, amplified with a 60°C annealing step; same reaction employed in quantitative PCR. (D) 104 bp *RpL32 *CDS fragment amplified with a 60°C annealing step; same reaction employed in quantitative PCR. (E) 193 bp *Hsp70 *fragment, from the 5' end of the CDS, amplified with primers specific to *Hsp70Aa/Ab*. (F) 193 bp *Hsp70 *fragment, from the 5' end of the CDS, amplified with primers specific to *Hsp70Ba/Bb/Bbb/Bc*.

We confirmed that the *Hsp70*^- ^strain lacked full-length genomic *Hsp70 *copies via individual-fly PCR (Figure 5). Amplification of a 1 kb fragment conserved in all genomic *Hsp70 *copies, corresponding to the 3' half of the 2 kb coding sequence (CDS), was unsuccessful off of *Hsp70*^- ^individual-fly DNA templates. The same reaction conducted off of DNA from all other strains produced a single band (Figure 5a). DNA of all strains yielded successful amplification of a 1 kb control fragment (Figure 5b). These results indicate that the *Hsp70*^- ^strain lacks *Hsp70 *gene copies of at least 1 kb, in agreement with the Southern blotting of Gong and Golic [13]. However, when we amplified the 140 bp *Hsp70 *fragment from the 3' end of the CDS used in quantitative PCR off of the individual-fly DNAs, a faint band was observable in *Hsp70*^- ^lanes (Figure 5c). Amplification of the *RpL32 *fragment produced bands of consistent intensity across strains (Figure 5d), indicating that the faint *Hsp70 *product in the *Hsp70*^- ^strain may result from amplification of a rare target. Raising the annealing temperature by 5°C further reduces the *Hsp70*^- ^band intensity, consistent with this idea (data not shown). We sequenced the 140 bp *Hsp70 *product obtained from an *Hsp70*^- ^individual, and found it was 100% identical to *Hsp70Aa*/*Hsp70Ab *CDS (data not shown).

To further establish the nature of this fragment in the *Hsp70*^- ^strain, we took advantage of divergent sites near the 5' end of the *Hsp70 *CDS. Positions 179 and 332 (relative to translation start) bear fixed nucleotide differences that differentiate *Hsp70Aa *and *Hsp70Ab *from *Hsp70Ba*, *Hsp70Bb*, *Hsp70Bbb *and *Hsp70Bc*. We designed primers whose 3' ends fell on these divergent sites to differentially amplify the otherwise ~96% identical CDSs. Amplification of this 193 bp fragment using the *Hsp70Aa*/*Hsp70Ab*-specific primers failed in all *Hsp70*^- ^and *Hsp70*^*A*-*Ba*- ^individuals but was successful in all *Hsp70*^+ ^and *Hsp70*^*traIII *^individuals (Figure 5e). Using the *Hsp70Ba*/*Hsp70Bb*/*Hsp70Bbb*/*Hsp70Bc*-specific primers resulted in successful amplification from individuals of all strains except *Hsp70*^- ^(Figure 5f). The above results suggest that a fragment of either *Hsp70Aa *or *Hsp70Ab *remains in the *Hsp70*^- ^genome, that the fragment consists of less than 1 kb of the 3' end of the 2 kb CDS and that this fragment is expressed. The *Hsp70 *deletions have neither been mapped to the nucleotide level nor confirmed via protein analysis [13,14]. Therefore, the full sequence, proximity to promoter(s) and protein-coding potential of the truncated *Hsp70 *product in the *Hsp70*^- ^strain remain unknown. Regardless of the source of the *Hsp70*^- ^short product, its expression levels are extremely low (Table 3). In the following, we consider strains' *Hsp70 *levels relative to their own control treatment values; therefore, *Hsp70 *levels in the *Hsp70*^- ^strain have no impact on measurement in any other strain.

*Hsp70 *expression after PT is positively associated with *Hsp70 *copy number in all strains, and is sensitive to both temperature and recovery time (Figure 2). Mixed model ANOVAs report highly significant strain, treatment and strain-by-treatment interaction effects at both zero and one hour post-treatment (Table 2). Figure 2 illustrates LSM estimates of *Hsp70 *expression, calculated by the mixed model ANOVAs, and fold changes at different treatments (within strain) relative to C levels. Induction immediately after PT, relative to C levels, ranges from 13- to 144-fold (Figure 2). After 1 h of recovery, *Hsp70 *PT induction relative to C is reduced and ranges from 3- to 72-fold. At both timepoints, levels of PT induction increase linearly with *Hsp70 *copy number.

HS and PT+HS treatments cause *Hsp70 *expression to fall to control levels or below control levels in the *Hsp70*^*A*-*Ba*-^, *Hsp70*^+ ^and *Hsp70*^*traIII *^strains. In the *Hsp70*^- ^strain, a slight increase in expression follows PT and PT+HS treatments (Figures 2a,b), likely representing expression of an imprecisely deleted *Hsp70Aa *or *Hsp70Ab *CDS (as discussed previously). The increase is consistent with the strong induction of other *Hsps *after PT+HS in this strain (see the discussion below and Figure 3). Overall, while PT strongly induces *Hsp70 *in the non-null strains to a degree consistent with their *Hsp70 *copy number, the other treatments do not induce *Hsp70*. These results are consistent with previous observations regarding *Hsp70 *expression over short timescales: thermal treatments of 30–37°C immediately induce *Hsp70*, but more severe temperatures do not [28].

### Expression of other genes

We examined the expression of seven constitutive stress-response genes (*GstE1*, *Hsc70-1*, *Hsc70-2*, *Hsc70-3*, *Hsc70-4*, *Hsc70-5 *and *Hsp60*) and ten inducible stress-response genes (*Hsp22*, *Hsp23*, *Hsp26*, *Hsp27*, *Hsp40*, *Hsp67Ba*, *Hsp67Bb*, *Hsp67Bc*, *Hsp68 *and *Hsp83*), immediately and 1 h after treatment. As indicated in Table 2, problematic genes were not considered: *Hsc70-1 *failed to amplify efficiently and was excluded from analysis. *Hsp67Bb *was not amplified with sufficient specificity and produced multiple PCR products. *Hsp67Ba *showed no significant strain, treatment or strain-by-treatment interaction effects immediately after treatment and was excluded from analysis. *Hsp67Bc *showed significant treatment and strain-by-treatment interaction effects; however, because the majority of the *Hsp67 *genes were excluded, we did not consider *Hsp67Bc *further (Table 2).

The remaining genes showed significant strain, treatment and strain-by-treatment interaction effects in the majority of cases, according to ANOVAs (Table 2). We classified these genes as inducible or constitutive based on whether they displayed upregulation following PT in a majority of strains. Neal et al [17] considered *Hsp40 *and *Hsp83 *as 'constitutive' and both genes are expressed in C conditions (Figure 3). However, both genes showed strong upregulation following PT and are coordinately expressed with the other inducible genes we examined (Figure 3). As above, we report each gene's LSM expression level, calculated by the mixed model ANOVAs, and express fold changes at different treatments relative to C levels (within strain; see Figures 4 and 5). Note that fold changes in Figures 4 and 5 reflect averages of the inducible and constitutive genes in each figure.

### Inducible genes

*Hsp22*, *Hsp23*, *Hsp26*, *Hsp27*, *Hsp40*, *Hsp68 *and *Hsp83 *display coordinate expression patterns within strains, but divergent patterns between *Hsp70*^- ^and the other strains (Figure 3). In the *Hsp70*^*A*-*Ba*-^, *Hsp70*^+ ^and *Hsp70*^*traIII *^strains, expression is upregulated at both zero and one hour after PT, with average fold induction relative to C ranging from 17.3- to 51.5-fold (Figures 3c–h). Expression following HS and PT+HS treatments returns to C levels or below, with the exception of *Hsp22 *and *Hsp26*, which are slightly upregulated after PT+HS39 in the *Hsp70*^+ ^and *Hsp70*^*traIII *^strains. Average fold expression relative to C for all seven genes at PT+HS39, at both timepoints, ranges from a decrease of 142.2-fold to an increase of 2-fold (Figures 3c–h). The above patterns are concordant with patterns of *Hsp70 *expression (Figures 2c–h).

The *Hsp70*^- ^strain displays a qualitatively different pattern of expression for the inducible genes in comparison with the other strains (Figures 3a,b). Expression of all seven genes is upregulated after PT to a level indistinguishable from the other strains; however, PT+HS treatments also cause upregulation. Expression is especially pronounced 1 h after PT+HS39 (e.g. 1227.4-fold, see Figure 3b). In contrast, the other strains show net downregulation after the PT+HS treatments.

### Constitutive genes

*Hsc70-2*, *Hsc70-3*, *Hsc70-4*, *Hsc70-5*, *Hsp60 *and *GstE1 *also display coordinate expression patterns in the *Hsp70*^*A*-*Ba*-^, *Hsp70*^+ ^and *Hsp70*^*traIII *^strains, and divergence in the *Hsp70*^- ^strain (Figure 4). Expression levels during control conditions vary among genes, but within strains, each gene's expression is generally not upregulated by PT (at either timepoint). In the *Hsp70*^*A*-*Ba*-^, *Hsp70*^+ ^and *Hsp70*^*traIII *^strains, HS causes downregulation of the constitutive genes, with the effect increasing with time: by 1 h post-treatment, average expression levels decrease 34.4- to 159.4-fold relative to C (Figures 4c–h). In the *Hsp70*^- ^strain, this effect is absent at HS39 and reversed at HS39.5, where average expression increases 26.6- and 12.1-fold (Figures 4a,b). PT+HS treatments cause further downregulation in the *Hsp70*^*A*-*Ba*-^, *Hsp70*^+ ^and *Hsp70*^*traIII *^strains at both timepoints, with the decrease ranging from 14.9- to 1047.2-fold (Figures 4c–h). In contrast, the *Hsp70*^-^strain shows net upregulation after PT+HS treatments. This effect is largely due to *Hsc70-3 *and *Hsc70-4*, which have lower C and PT expression levels in the *Hsp70*^- ^strain than in the other strains, and rise after PT+HS instead of falling (Figures 4a,b).

## Discussion

**Figure 6:**
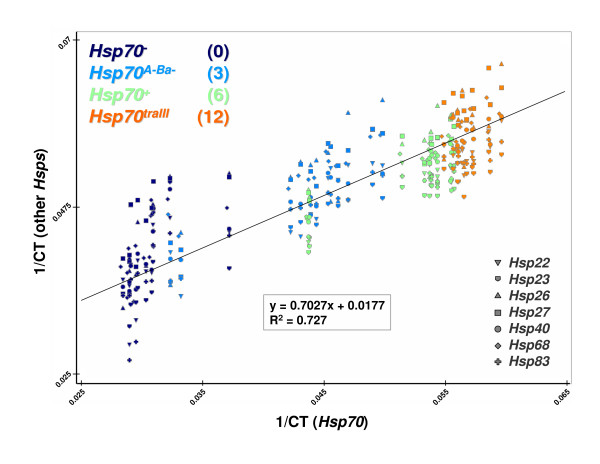
**Reduced major axes regression of inducible *Hsp *gene expression on *Hsp70 *gene expression**. Graph charts raw gene expression values (reciprocal CT scores) of *Hsp70 *(*x*-axis) versus seven inducible *Hsp*s (*y*-axis). Symbol colors indicate strain; the legend in the top left denotes strain, with *Hsp70 *copy number in parentheses. Symbol shapes indicate gene; see the legend in the lower right. Regression line in black; equation and *R*^2 ^values in box.

Our results indicate that *Hsp70 *loss causes alterations of both inducible and constitutive stress gene expression that are ultimately insufficient for inducible tolerance of severe heat shock. In addition, we find that both increasing *Hsp70 *copy number and *Hsp70 *expression are associated with increases in the expression of multiple inducible *Hsp *genes (see the discussion in the following and Figure 6). We now discuss the implications of these results for stress tolerance, compensatory gene expression and the regulation of *Hsp *gene expression.

### Thermotolerance

Inducible thermotolerance is severely curtailed in the *Hsp70*^- ^strain and increases linearly with *Hsp70 *copy number in the other strains. The effect is especially pronounced at 39.5°C (Figure 1). These results are consistent with previous research [14,15,29], but highlight the extreme sensitivity of thermotolerance traits to assay temperature. Furthermore, the strong differences between basal and inducible thermotolerance among the strains reflect the potent effect of thermal pretreatment and heat shock protein induction. Previous research reported the maintenance of some degree of thermotolerance in the *Hsp70*^- ^strain, when assayed at lower heat shock temperatures and different heat shock durations that may not maximize the input of *Hsp70 *(e.g. 37°C [14]). Here, we designed our thermotolerance assays to maximize differences in survival among strains varying in *Hsp70 *copy number and expression and have found the *Hsp70*^- ^strain almost completely deficient in basal and inducible tolerance of severe heat shock (Figure 1). We measured gene expression in animals undergoing the same severe thermal treatments and interpreted differences among the strains accordingly.

### Analysis of gene expression

When measuring gene expression via qrtPCR, researchers typically express candidate gene levels relative to a control gene, often a 'housekeeping' gene that is ubiquitously expressed independently of experimental treatments. This adjustment serves to control for variation in RNA/cDNA extractions. Rather than express our measurements in candidate gene:control gene ratios, here we estimate each gene's own expression level according to an ANOVA model which includes expression of the *RpL32 *control gene as a covariate. The model also includes effects imparted by both experimental error (extraction, PCR replicate) and the biology we wish to explore (strain, treatment, timepoint). *RpL32*, strain and treatment all have a significant effect on candidate gene expression (see Table 2), indicating that these factors, rather than error, explain much of the variation in gene expression.

Does the fact that *RpL32 *expression is itself affected by the treatments we employed have an impact on the analysis? Specifically, *RpL32 *expression decreases with HS and PT+HS treatments across strains. Notwithstanding a sensible biological interpretation of this observation (severe stress downregulates housekeeping genes and upregulates heat shock genes), we would predict that if *RpL32 *was a more important factor in candidate gene expression than strain or treatment, our model would perform poorly during HS and PT+HS and, thus, generate LSM estimates that do not match raw expression values.

We recalculated the expression curves presented in Figures 3 and 4 using raw data (CTs) rather than LSMs and found the opposite. The curves are given as Additional file 1 and Additional file 2 and are strikingly concordant with the model outputs illustrated in Figures 3 and 4. The effects of treatment and strain on *Hsp *gene expression levels are evident whether considering modeled or raw data. This indicates that the biological signals (e.g. upregulation of inducible *Hsps *following thermal pretreatment, differential regulation in the *Hsp70*^- ^strain) are stronger than the 'noise' imparted by variation in control gene expression and/or experimental error.

### Inducible gene expression

The expression levels of seven inducible *Hsp *genes after PT, relative to C levels, are high in all of the strains we examined but are not strongly associated with *Hsp70 *copy number (Figure 3). However, when considered in isolation, C and PT expression levels appear to increase with *Hsp70 *copy number (top to bottom in Figure 3). Furthermore, *Hsp70 *expression clearly increases with *Hsp70 *copy number when expressed either in absolute or PT-relative-to-C terms (Figure 2). Finally, the housekeeping *RpL32 *gene levels remain a highly significant covariate in every inducible gene/timepoint combination but two (Table 2). This indicates that the distribution of error associated with variation in RNA/cDNA manufacture is not unevenly associated with any strain, gene or timepoint, and that direct comparison of absolute inducible gene expression levels (CTs) is possible. We therefore explored whether the highest absolute inducible *Hsp *expression levels in each strain, those produced by PT, could be predicted by corresponding PT *Hsp70 *expression levels. Since the expression levels of *Hsp70 *and the other *Hsps *each have associated error, simple linear regression is inappropriate for exploring this relationship. As such, we conducted reduced major axes regression using the software of K Van der Linde [30]. We pooled raw zero- and one-hour PT expression data (reciprocal CT values) within each strain and regressed the other *Hsps *on *Hsp70*.

We find that after PT, *Hsp70 *expression is a strong predictor of other inducible *Hsp *expression (*R*^2 ^= 73%; see Figure 6). The four strains cluster on both axes, indicating that increasing *Hsp70 *copy number is strongly associated with both measures of gene expression. That multiple *Hsp *genes would show coordinate upregulation following thermal pretreatment is not surprising: the inducible *Hsp *genes were first noticed as a suite of heat-induced transcriptional puffs and their coexpression is well established [31,32]. We thus might expect that strains or species that vary in the amount or activity of a known global stress-response regulator, such as HSF, could show coordinate alteration of *Hsp *gene expression levels. These strains, however, differ only in *Hsp70 *copy number and *Hsp70 *expression. This indicates that the *Hsp70 *gene or Hsp70 protein has coordinate effects on other *Hsp *expression, and that increasing *Hsp70 *levels in turn upregulate the other *Hsps *(Figure 6). A broad effect of *Hsp70 *on the regulation of other *Hsps *is consistent with previous research, but the positive direction of the effect is unexpected.

In many systems, free Hsp70 protein can bind to HSF and prevent transactivation that stimulates *Hsp *transcription, thereby negatively regulating Hsp levels in a classic feedback loop [33,34]. Consistent with this model, overexpression of Hsp70 protein in the absence of stress can repress inducible transcription of *Hsp *genes [35-37]. However, our results indicate that increasing *Hsp70 *gene expression levels are associated with increases in the expression of other *Hsp *genes, at least over brief timescales. This unexpected finding hints at additional mechanisms of *Hsp *transcriptional regulation independent of Hsp70/HSF protein interaction, and requires further research. An intriguing possibility involves a candidate 'cost' of Hsp70 expression in the absence of severe heat shock [11,12]. Hsp70 is a generalist chaperone that, in the absence of thermally denatured protein substrates to bind, could instead bind diverse proteins and pull them from their native conformations. These newly non-native proteins could in turn induce a further stress response, including the upregulation of additional *Hsp *genes. This misregulation would be especially deleterious when Hsp70 is overexpressed, such as in the *Hsp70*^*traIII*^strain, and when thermal conditions strongly induce Hsp70 but do not precede a severe heat shock, such as the PT treatment we employed. Clearly, existing and future studies of stress induced gene expression in *Drosophila*, especially those examining global transcriptional responses in mutant or selected strains that may vary in *Hsp70 *expression [17,38], should be examined for coordinate upregulation of other *Hsps*.

In contrast to the association between levels of *Hsp70 *and the other inducible *Hsp *genes after PT, the inducible *Hsps *are upregulated after PT+HS in the *Hsp70*^- ^line. This result is clearly not explained by increasing *Hsp70 *copy number. It is, however, consistent with increased thermal damage in the *Hsp70*^- ^strain and/or improper repression of the heat shock response. Thermally denatured proteins are a primary stimulus of the heat shock response [39]. Since *Hsp70*^- ^larvae fail to survive PT+HS, perhaps the increase in *Hsp *expression simply reflects that the severity of stress combined with lack of *Hsp70 *causes extensive *Hsp-*inducing protein damage (from which the animals are ultimately unable to recover). Alternatively, the lack of *Hsp70 *could cause failure of proper *Hsp *transcriptional attenuation post-stress. This hypothesis was favored by Gong and Golic [14], who applied a brief, mild heat shock to the *Hsp70*^-^strain and observed transcriptional *Hsp *'puffing' on polytene chromosomes that persisted longer than that observed in a wild-type strain. We did not observe upregulation of the inducible *Hsps *after HS in the *Hsp70*^- ^strain, even though survival of HS and PT+HS is equally low. This may indicate that over the short timespan in which we examined gene expression, PT+HS is more damaging than HS (in the absence of *Hsp70*). To explore whether HS eventually upregulates the inducible *Hsps *in the *Hsp70*^- ^strain, future research will examine patterns of gene expression, thermal tissue damage and cellular damage indicators such as protein aggregates and ubiquitin conjugates at additional, extended timepoints. Whether the *Hsp *upregulation in the *Hsp70*^- ^strain is a result of damage and/or a lack of transcriptional repression is an open question. Regardless of the mechanism of *Hsp *upregulation after PT+HS in the *Hsp70*^- ^strain, the response is clearly not sufficient to generate increased inducible thermotolerance (Figure 1).

### Constitutive gene expression

As was seen with the inducible *Hsps*, the constitutive genes display coordinate regulation (Figure 4). The direction of regulation, however, is different: the constitutive genes are expressed at low levels after C and PT, and rise with HS39.5 and both PT+HS treatments in the *Hsp70*^- ^strain, as opposed to falling from higher C and PT levels to low HS and PT+HS levels in the other strains. Furthermore, *Hsp70 *levels do not predict levels of the constitutive genes to the degree they did for the inducible genes. These results indicate that *Hsp70 *may be more uncoupled from the regulation of the constitutive genes. Neal et al [17] reported upregulation of constitutive stress gene expression in *HSF *mutant *Drosophila *and suggested that such upregulation could compensate for the loss of inducible *Hsp *expression. Our results suggest that disrupting *Hsp70 *specifically, instead of the entire inducible *Hsp *response through *HSF*, induces similar upregulation of constitutive genes while also influencing the expression of other inducible *Hsps*. Again, however, the modification of gene expression caused by *Hsp70 *loss does not provide compensatory thermotolerance in our assays.

We identified genes whose expression is altered by thermal stress in *Hsp70 *mutant backgrounds: genes that did not provide compensatory thermotolerance in our assays. Future experiments will determine whether inducing any of these genes prior to stress application will promote thermotolerance in the *Hsp70*^- ^strain. Given the strong effect of *Hsp70 *modification on inducible and constitutive *Hsp *expression, determining whether and how any of these candidate stress protective genes can operate independently of Hsp70 and its associated costs remains a challenge.

## Conclusion

*Hsp70 *copy number is strongly associated with thermotolerance, with the *Hsp70*^- ^strain displaying virtually zero basal or inducible tolerance of 39°C and 39.5°C. *Hsp70 *copy number is also strongly positively associated with thermal-pretreatment induction levels of *Hsp70 *and seven other inducible *Hsp *genes, suggesting an upregulatory effect of *Hsp70 *on the stress-induced transcriptional response. The *Hsp70*^- ^strain displays qualitatively different patterns of gene expression after severe heat stress, including upregulation of inducible and constitutive *Hsps*. Since these alterations do not produce increased thermotolerance in the *Hsp70*^- ^strain, they do not represent genetic mechanisms of phenotypic compensation, but suggest candidate genes for prophylactic overexpression.

## Methods

### *Drosophila *strains and nomenclature

Wild-type *Drosophila melanogaster *possess five or six *Hsp70 *genes distributed at two genomic loci, depending on strain: *Hsp70Aa*, *Hsp70Ab*, *Hsp70Ba*, *Hsp70Bb*, *Hsp70Bbb *and *Hsp70Bc *[40,41]. All four strains used in this study originated from a common six-*Hsp70 *genetic background (*w*^1118^). The cisIII and traIII strains, henceforth *Hsp70*^+ ^and *Hsp70*^*traIII*^, were donated by Martin Feder (University of Chicago). These sister strains are identical except for a transgenic insertion of six *Hsp70 *genes on the third chromosome of *Hsp70*^*traIII *^(*Hsp70*^+ ^has six *Hsp70 *genes [15]). The *Hsp70*^*A*-*Ba*- ^and *Hsp70*^- ^strains, obtained from the Bloomington *Drosophila *stock center, possess three and zero genomic copies of *Hsp70*, respectively [13]. In the *Hsp70*^*A*-*Ba*^strain, the *Hsp70Aa*, *Hsp70Ab *and *Hsp70Ba *genes have been deleted; in the *Hsp70*^- ^strain, all *Hsp70 *genes have been deleted. The *Hsp70*^- ^and *Hsp70*^*A*-*Ba*- ^strains were re-isogenized via crossing with *w*^118^; *D*^3^*/TM3Ser *balancer-chromosome flies and subsequent sib mating of *Hsp70 *mutant/*TM3Ser *heterozygous individuals. All strains were reared on semi-defined medium [42] in standard vials at 22°C and a 12 h:12 h light:dark cycle.

### Thermotolerance

Larvae for thermotolerance assays were reared as follows. Population cages, approximately 4 l in volume, were constructed of large diameter PVC pipe, plexiglass and nylon stockings. Between 200 and 300 adults of each strain were introduced to cages maintained at 22°C as above. Each cage received a standard Petri plate containing 50 ml of media supplemented with live yeast. Every 24 h, plates were removed, swapped with fresh plates, covered loosely and incubated at 22°C. This regime produced plates containing 500–1500 developmentally synchronized larvae. Under- or over-crowded plates were not used.

Larvae were extracted from the plates when third instar larvae were first observable on the food surface and plate lid. Briefly, third-instar larvae were floated out of the media using salt water, removed via filtration on Whatman paper and rinsed in distilled water. Batches of 20 larvae were gently transferred with paintbrushes from the filter paper to standard glass rearing vials containing 15 ml of instant *Drosophila *media (Carolina Scientific Co.) supplemented with live yeast. Vials were plugged with rubber stoppers and placed at 22°C for approximately 30 min to allow larvae to recover. Thermotolerance assays were conducted over several days; on each individual day, vials were randomly assigned to six treatments (multiple treatments per day) according to a random block design. A minimum of eight vials per strain/treatment/temperature combination were prepared and analyzed. Treatments were conducted and synchronized over 3 h spans as follows.

### Treatments

We designed thermal treatments that maximized differences in inducible thermotolerance among the *Hsp70*^-^, *Hsp70*^*A*-*Ba*-^, *Hsp70*^+ ^and *Hsp70*^*traIII *^strains. Previous work established that thermal pretreatment at 36°C induces near-maximal Hsp70 expression with minimal mortality in wild-type strains [28]. Heat shocks of 39°C and 39.5°C, with and without thermal pretreatment, were administered (to measure basal and inducible thermotolerance, respectively). Gong and Golic [14] report defects in thermotolerance of 39°C in the *Hsp70*^- ^strain; we also employed 39.5°C because the more severe heat shock caused the greatest differences in inducible thermotolerance among all four strains and produced the strongest differentiation between basal and inducible thermotolerance (Figure 1).

All vials were inverted and placed in wire racks. Control ('C') vials were then placed in a 22°C incubator for 3 h. Pretreatment ('PT') vials were submerged in a circulating water bath at 36°C for 1 h, then removed and placed in a 22°C incubator for 2 h. Heat shock ('HS') vials were placed in a 22°C incubator for 2 h then submerged in a circulating water bath at either 39°C or 39.5°C for 1 h. Pretreatment plus heat shock ('PT+HS') vials received 1 h pretreatment as above, followed by 1 h at 22°C, then 1 h heat shock at 39°C or 39.5°C as above.

Following treatment, stoppers were carefully removed and replaced with cotton plugs, and any larvae on the stoppers were gently transferred to the vial wall with a paintbrush. Vials were then placed upright in a 22°C incubator for continued larval development. Survival was scored as the number of successfully eclosed adults, measured two days after first observed eclosion (per strain/treatment combination) and three days subsequently. Binary logistic regression of thermotolerance (survival) data examined strain, treatment and strain-by-treatment interaction effects (Table 1).

### Gene expression

Larvae for gene expression analysis were reared, selected and extracted as above. Treatments (C, PT, HS and PT+HS) were as above, with two differences: larvae were collected in groups of 15 and placed in RNAse-free 1.5 ml microfuge tubes containing 50 μl of sterilized 50% w/v light corn syrup in DEPC-treated water. Tubes were submerged in circulating water baths and/or recovered in incubators as above. We measured gene expression immediately and 1 h after treatment, because *Hsp *mRNAs in particular display rapid turnover depending upon the continuation or cessation of stress conditions [16,43]. Accordingly, immediately following treatment or after 1 h of recovery at 22°C ('zero hour' and 'one hour', respectively), tubes were submerged in liquid nitrogen to flash freeze larvae and then stored at -80°C. A minimum of four tubes per strain/treatment/temperature/timepoint combination were prepared.

RNA extraction and complementary DNA ('cDNA') synthesis: Total RNA was extracted from each group (tube) of 15 larvae according to the Trizol-based protocol of Fiumera et al [44]. Following extraction, RNA was resuspended in 50 μl sterile DEPC-treated water and stored at -80°C. Synthesis of cDNA again followed [15]; briefly, 4 μl total RNA per synthesis reaction was treated with DNAse to remove genomic DNA, primed with oligo-dT and incubated with reverse transcriptase and RNAse inhibitor according to the manufacturer's protocols (Promega Inc.). Following synthesis, cDNA was diluted 1:15 with sterile water and stored at -80°C.

### Selection of genes and PCR primers

We focused on known (annotated) *Drosophila melanogaster *constitutive and inducible heat shock genes in addition to *Hsp70 *(see Table 2 and [16]). For each gene, mRNA sequence was downloaded from FlyBase [45]; for genes with multiple transcripts, only consensus sequences contained in all splice forms were considered. To reduce variability in apparent transcript abundance owing to non-perfect processivity of the reverse transcriptase, we designed primers based on the 3' ends of genes. Primers were designed using default settings of the Primer3 package [46].

For *Hsp70*, primers were designed to amplify a 140 bp fragment of both *Hsp70A *and *Hsp70B *CDSs. All genomic *Hsp70 *copies are invariant at the lower primer site and vary at one internal position in the upper primer site. Control qrtPCR reactions conducted on cDNA prepared from strains bearing only the *Hsp70A *or *Hsp70B *loci found no significant difference in amplification efficiency (data not shown).

For all other genes, an upper primer was designed to match the 3'-most coding region and a lower primer to match the 3'-UTR of each gene, to amplify a 100–150 bp product. This strategy also ensured specificity of amplification, as the 3'-UTRs of most *Hsp *genes are divergent (even when CDSs are highly similar [40]). Primers for all genes are available upon request. To amplify a standard for measurement of relative transcript abundance, we designed similar primers specific to a 104 bp region of the ubiquitously expressed *RpL32 *ribosomal protein gene [44].

### Quantitative real-time PCR

Three cDNAs per strain/treatment/timepoint combination were used as template for qrtPCR. Each cDNA was distributed in duplicate onto two of four master 96-well plates according to a random block design. Twenty replicate PCR plates were made from each master plate, with 5 μl of cDNA in each well, and frozen at -20°C for future use. QrtPCR was conducted on a BIO-RAD MyIQ thermocycler, according to the manufacturer's instructions and using BIO-RAD SYBR-green reagents. Reaction conditions for all genes were: 2 min at 94°C, 40 cycles of 30 s at 92°C, 30 s at 60°C and 30 s at 72°C, and a final extension of 5 min at 72°C. Following amplification, a thermal disassociation protocol verified the production of single PCR products. For each gene amplification, a serial dilution analysis was also performed to calculate reaction (primer) efficiency and reaction kinetics. Reactions producing multiple or inconsistent products and/or efficiencies of less than 75% were not included in analysis (see Table 2). BIO-RAD MyIQ software was used to calculate the critical threshold (CT) for each reaction and tabulate results. Statistical treatment and analysis of qrtPCR data is detailed in the results section.

### Confirmation of *Hsp70 *deletion

To confirm whether the *Hsp70 *genes were deleted from the *Hsp70*^- ^strain, we extracted DNA from individual flies of all strains according to Gloor et al [47]. From these preparations, we conducted standard PCR to amplify a number of products. First, we amplified a 1 kb fragment of *Hsp70*, corresponding to the 3' half of the ~2 kb CDS, conserved in all genomic *Hsp70 *copies [28]. Second, we amplified a 1 kb fragment of the *fd3f *gene for verification of DNA preparation quality (primers available upon request). Next, we amplified both the 140 bp *Hsp70 *and 104 bp *RpL32 *gene fragments used in quantitative PCR, using the same reaction conditions as above.

## List of abbreviations

ANOVA, analysis of variance; C, control; CDS, coding sequence; CT, critical threshold; HS, heat shock; Hsc: heat shock cognate (*italics*, gene; non-italics, protein); HSF, heat shock transcription factor; Hsp, heat shock protein (*italics*, gene; non-italics, protein); LSM, least square mean; PCR, polymerase chain reaction; PT, pretreatment; PT+HS, pretreatment plus heat shock; qrtPCR, quantitative real-time PCR

## Authors' contributions

BRB conceived of and designed the study, conducted fly crosses, conducted standard PCRs, conducted statistical analyses and drafted the manuscript. CCH conducted RNA and cDNA synthesis, designed and conducted the cDNA plating scheme, conducted quantitative and standard PCRs and assisted with drafting the manuscript. MN conducted RNA and cDNA synthesis and conducted quantitative and standard PCRs. BWD assisted in designing the study, conducted quantitative PCRs and assisted with statistical analyses. All authors participated in thermotolerance experiments and read and approved the manuscript.

## Supplementary Material

Additional file 1 **Supplemental Figure 1: Variation in raw inducible *Hsp *gene expression**. Each graph displays seven genes' expression following C, PT, HS39, HS39.5, PT+HS39, and PT+HS39.5 treatments (see Methods for full treatment description). Values expressed are inverse critical-thresholds (50 – cycle number). Symbols are means ± 1 S.E.; legend at top of figure indicates gene-symbol pairs. Graphs are organized according to strain (left to right) and timepoint post treatment (top to bottom). Click here for file

Additional file 2 **Supplemental Figure 2: Variation in raw constitutive *Hsp *gene expression**. Each graph displays six genes' expression following C, PT, HS39, HS39.5, PT+HS39, and PT+HS39.5 treatments (see Methods for full treatment description). Values expressed are inverse critical-thresholds (50 – cycle number). Symbols are means ± 1 S.E.; legend at top of figure indicates gene-symbol pairs. Graphs are organized according to strain (left to right) and timepoint post treatment (top to bottom). Click here for file
