# Supplementary figures and images for: Inducible and constitutive heat shock gene expression responds to modification of Hsp70 copy number in Drosophila melanogaster but does not compensate for loss of thermotolerance in Hsp70 null flies
Source: BMC Biol. 2008 Jan 22;6:5. doi: 10.1186/1741-7007-6-5 (PMC2257928; doi:10.1186/1741-7007-6-5)

**RAW (Inducible):** ◆ *Hsp22* ▲ *Hsp26* ✱ *Hsp40* ■ *Hsp23* ● *Hsp27* ▲ *Hsp68* ■ *Hsp83*

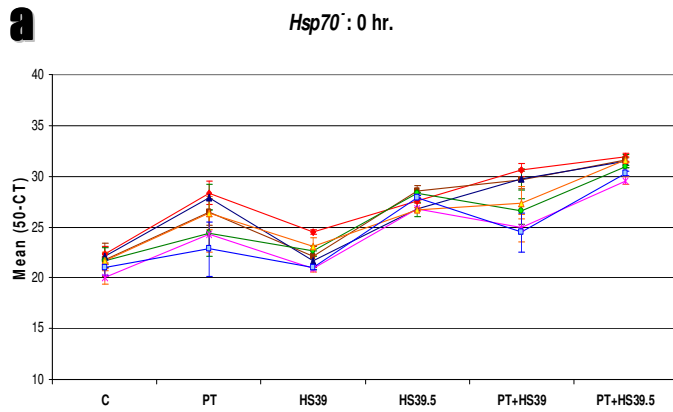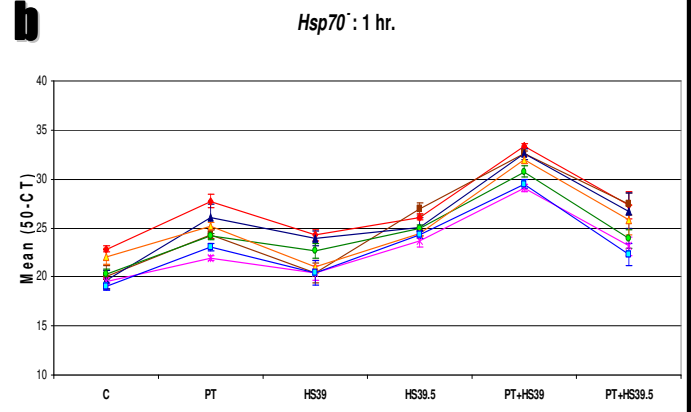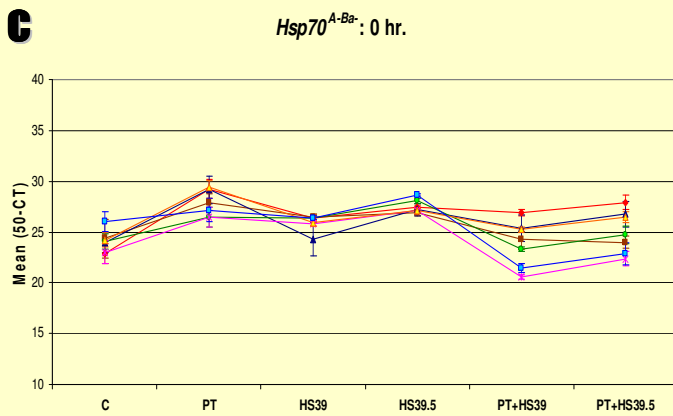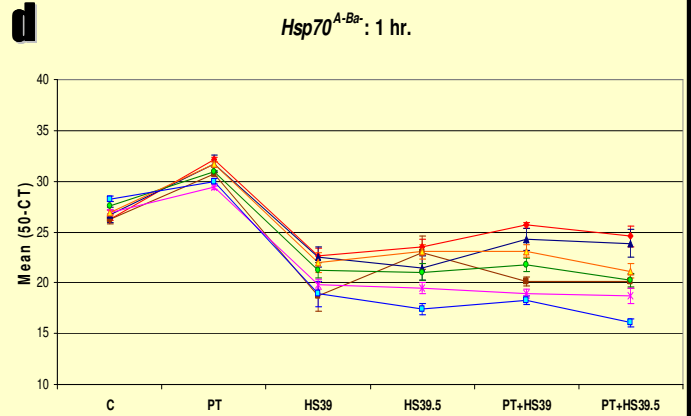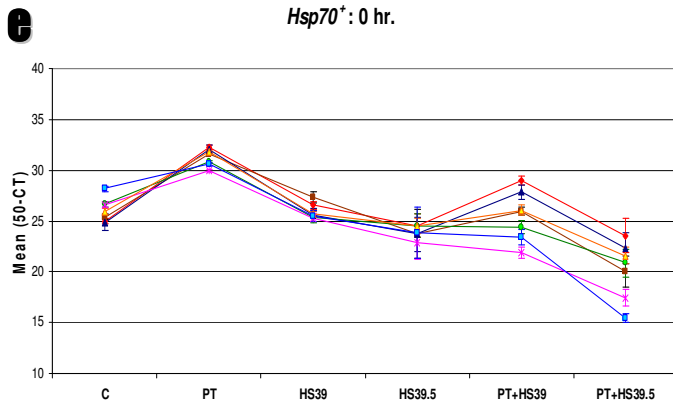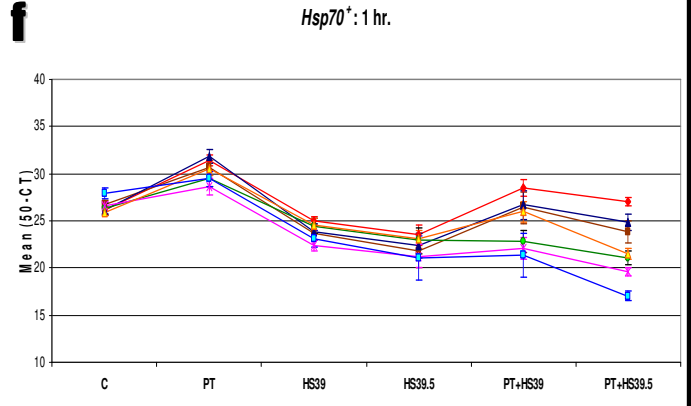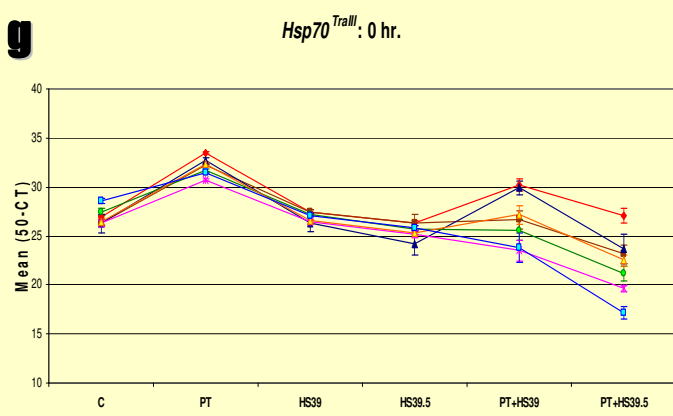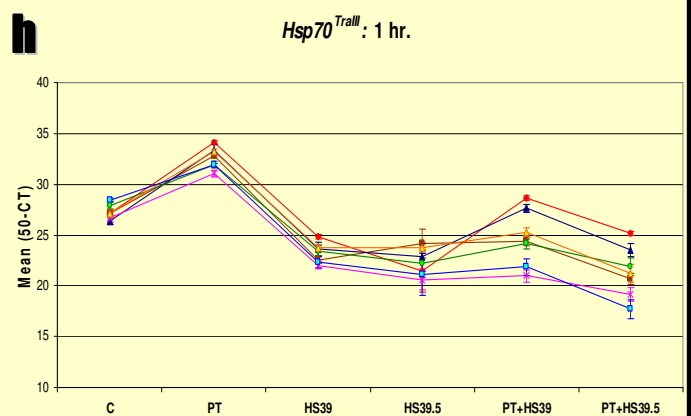

Supplement: Additional file 1 — Supplemental Figure 1: Variation in raw inducible Hsp gene expression. Each graph displays seven genes' expression following C, PT, HS39, HS39.5, PT+HS39, and PT+HS39.5 treatments (see Methods for full treatment description). Values expressed are inverse critical-thresholds (50 – cycle number). Symbols are means ± 1 S.E.; legend at top of figure indicates gene-symbol pairs. Graphs are organized according to strain (left to right) and timepoint post treatment (top to bottom). [file 1741-7007-6-5-S1.pdf]

# RAW (Constitutive):

◆ *Hsc70-2*

■ *Hsc70-3*

▲ *Hsc70-4*

✕ *Hsc70-5*

● *Hsp60*

● *GstE1*

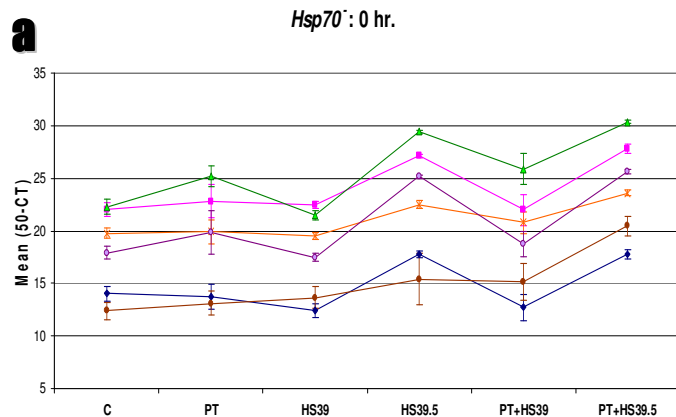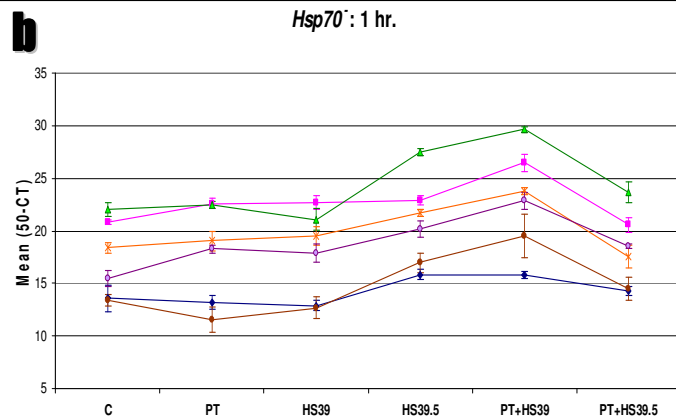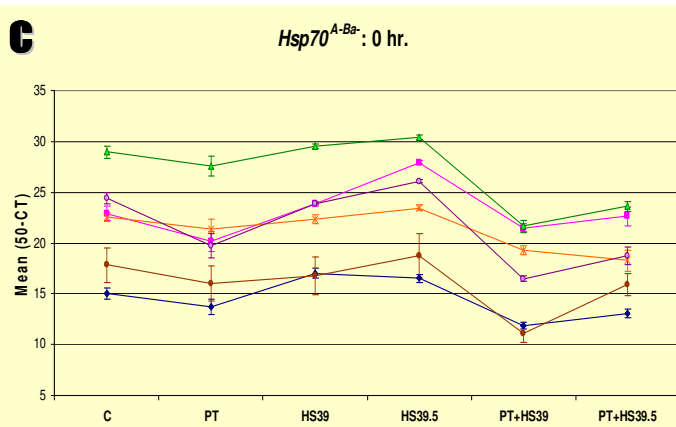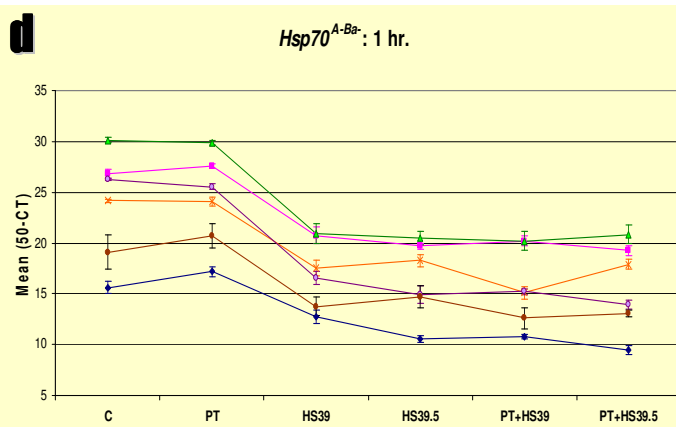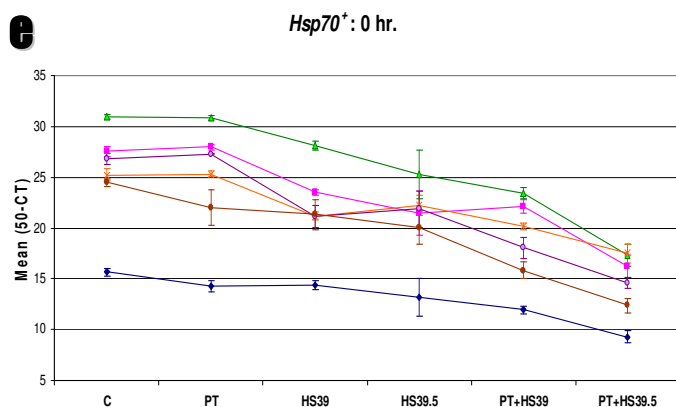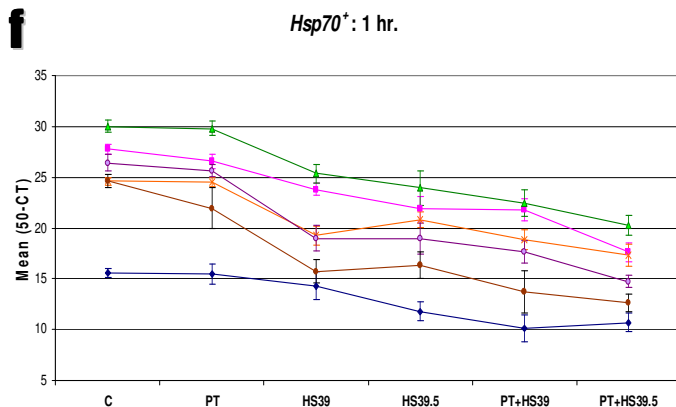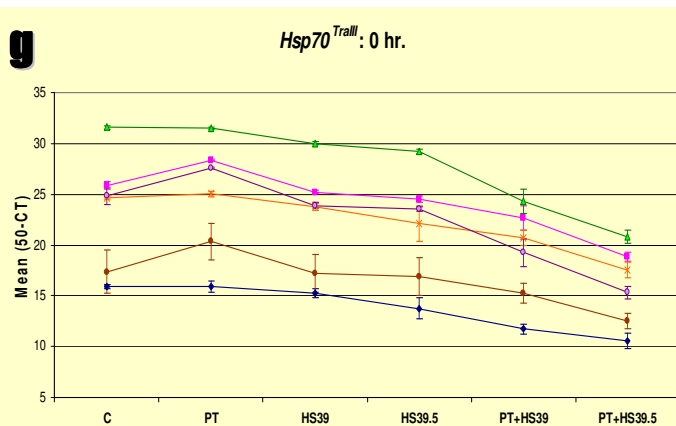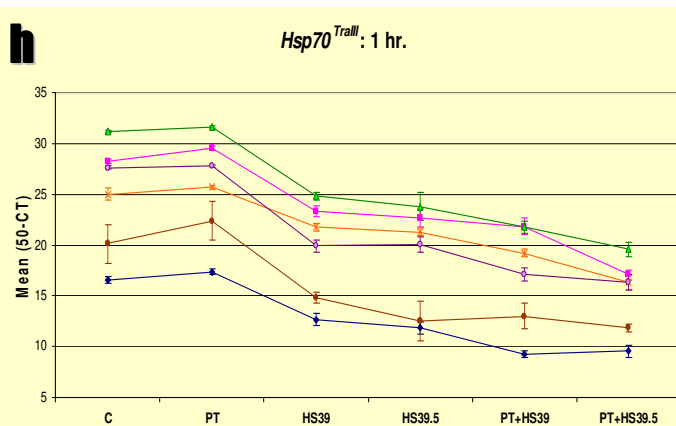

Supplement: Additional file 2 — Supplemental Figure 2: Variation in raw constitutive Hsp gene expression. Each graph displays six genes' expression following C, PT, HS39, HS39.5, PT+HS39, and PT+HS39.5 treatments (see Methods for full treatment description). Values expressed are inverse critical-thresholds (50 – cycle number). Symbols are means ± 1 S.E.; legend at top of figure indicates gene-symbol pairs. Graphs are organized according to strain (left to right) and timepoint post treatment (top to bottom). [file 1741-7007-6-5-S2.pdf]
